# Rem2, a member of the RGK family of small GTPases, is enriched in nuclei of the basal ganglia

**DOI:** 10.1038/srep25137

**Published:** 2016-04-27

**Authors:** Daniel J. Liput, Van B. Lu, Margaret I. Davis, Henry L. Puhl, Stephen R. Ikeda

**Affiliations:** 1 Laboratories of Molecular Physiology, National Institute on Alcohol Abuse and Alcoholism, National Institutes of Health, Bethesda, Maryland, 20892-9411, USA; 2 Integrative Neuroscience, National Institute on Alcohol Abuse and Alcoholism, National Institutes of Health, Bethesda, Maryland, 20892-9411, USA.

## Abstract

Rem2 is a member of the RGK subfamily of RAS small GTPases. Rem2 inhibits high voltage activated calcium channels, is involved in synaptogenesis, and regulates dendritic morphology. Rem2 is the primary RGK protein expressed in the nervous system, but to date, the precise expression patterns of this protein are unknown. In this study, we characterized Rem2 expression in the mouse nervous system. In the CNS, *Rem2* mRNA was detected in all regions examined, but was enriched in the striatum. An antibody specific for Rem2 was validated using a Rem2 knockout mouse model and used to show abundant expression in striatonigral and striatopallidal medium spiny neurons but not in several interneuron populations. In the PNS, Rem2 was abundant in a subpopulation of neurons in the trigeminal and dorsal root ganglia, but was absent in sympathetic neurons of superior cervical ganglia. Under basal conditions, Rem2 was subject to post-translational phosphorylation, likely at multiple residues. Further, *Rem2* mRNA and protein expression peaked at postnatal week two, which corresponds to the period of robust neuronal maturation in rodents. This study will be useful for elucidating the functions of Rem2 in basal ganglia physiology.

Rem2 is a member of the Rrad/Rem1/Rem2/Gem-Kir (RGK) subfamily of proteins, which in turn belong to the RAS superfamily of small GTPases. RGK proteins inhibit the function of high-voltage-activated (HVA) calcium channels by forming a non-conducting channel at the plasma membrane, interfering with channel gating, and disrupting channel trafficking^1^,^2^,^3^,^4^. Although most research has focused on the mechanisms of RGK protein inhibition of HVA calcium channels, RGK proteins have other cellular roles. In particular, Rem2 promotes the development of excitatory and inhibitory synapses, regulates dendritic spine densities, and is involved in shaping the dendritic arbor^5^,^6^,^7^,^8^. Thus, Rem2 may be necessary for proper development of the nervous system^9^ and participate in neurotransmission and neuroplasticity in the mature organism.

Although RGK proteins display some GTPase activity, several lines of evidence suggest that cycling between GTP- and GDP-bound states may not be the canonical mechanism regulating their activity^1^. For example, the RGK proteins display low intrinsic GTPase activity compared to typical RAS proteins^10^,^11^,^12^, which likely results from amino acid substitutions at residues critical for GTP hydrolysis^2^. Additionally, conformational changes in the switch domains of Rem2 and Rrad between GTP and GDP bound states are minimal^10^. Thus alternative mechanisms that regulate RGK activity have been sought^1^. In neurons, *Rem2* mRNA is upregulated by depolarization suggesting expression level modulation by neuronal activity^5^. Additionally, the phosphorylation state of Rem2 is a potential determinate of Rem2 function. Multiple consensus sites for protein kinases, including CaMKII and PKA^5^,^13^, are present in the N- and C-terminal regions of Rem2 and mutagenesis studies suggest that these sites are important for some Rem2 functions^6^,^8^. Moreover, phosphorylation of heterologously expressed Rem2 promotes association with 14-3-3 proteins, changing subcellular localization and function^6^,^13^.

Most of the research on RGK proteins has focused on the molecular mechanisms of RGK-HVA calcium channel interactions in heterologous expression systems or have relied on molecular approaches such as *in vitro* RNA interference. Although this work is essential to our understanding of the RGK protein family, there are only a few studies exploring the role of these proteins in the physiology of intact model organisms. Moreover, there is a paucity of information on which tissues and cell-types express RGK proteins. Rem2 appears to be the predominant family member expressed in the nervous system^11^,^14^,^15^; although two studies have shown that Gem is present in some neuronal populations^16^,^17^. One study found relatively high expression of *Rem2* transcript in the striatum and extended amygdala using *in situ* hybridization^18^, but did not examine Rem2 protein expression, identify cell-type expression, or determine subcellular localization. Such information is necessary for elucidating the functions of Rem2 in nervous system physiology.

In this study, we characterized the expression patterns of Rem2 throughout the mouse nervous system. *Rem2* mRNA was detectable in most nervous system tissues examined, however in the CNS *Rem2* mRNA and protein was enriched in nuclei of the basal ganglia. In the striatum, Rem2 immunoreactivity was restricted to medium spiny neurons (MSNs) and evidence suggests that Rem2 is phosphorylated at multiple residues. Developmentally, *Rem2* mRNA expression peaked at PND7-14, a time frame when dendritic spines and synapses are rapidly developing, implicating Rem2 in these events *in vivo*.

## Results 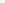


### Characterization of Rem2 mRNA and protein expression in the nervous system

**Figure 1:**
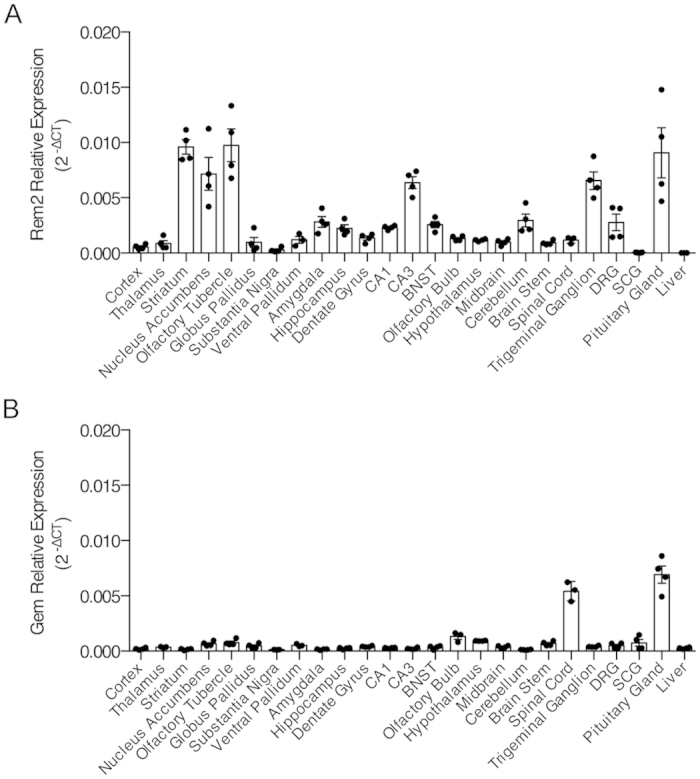
*Rem2* and *Gem* mRNA expression in the nervous system. Relative expression of *Rem2* mRNA (**A**) and *Gem* mRNA (**B**) across select regions of the nervous system. mRNA was quantified by qPCR and *Rem2* and *Gem* gene expression was normalized to *Actb*. Liver samples served as a negative control.

Previous reports have found that *Rem2* mRNA is primarily expressed in the brain^11^,^18^. However these studies did not provide a detailed description of transcript expression patterns within the CNS or report any information on Rem2 protein expression. Therefore, we sought to extend the available information on *Rem2* mRNA and protein expression in the nervous system. First, we isolated 24 regions within the central and peripheral nervous system and quantified *Rem2* mRNA using qPCR (Fig. 1A). *Rem2* mRNA was detectable in every region examined; however transcript levels were most abundant in regions of the striatum, CA3 region of the hippocampus, trigeminal ganglion and pituitary gland. Although Rem2 is thought to be the only RGK protein expressed in nervous system tissue, some reports have suggested that Gem has a physiological role in some neuronal populations^16^,^17^. Therefore, we quantified *Gem* mRNA in the same tissue arrays as above (Fig. 1B). *Gem* transcript was detected within a 40-cycle cutoff in all regions analyzed, but relative expression levels were very low except in the spinal cord and pituitary gland.

**Figure 2:**
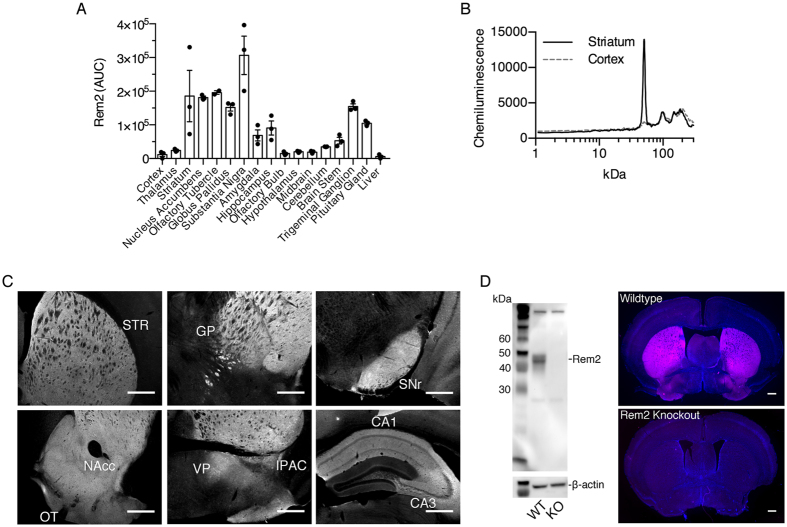
Characterization of Rem2 protein expression in the nervous system. (**A**) Rem2 protein expression in select regions of the nervous system. Rem2 was quantified using capillary electrophoresis followed by immunochemistry based detection (see materials and methods for details). (**B**) Representative electrophoretograms of samples for striatum and cortex showing high and low relative Rem2 signal. Data points in (**A**) were calculated by integrating the signal peak at approximately 50 kDa, which corresponds to Rem2 protein. (**C**) Representative images of Rem2 immunofluorescence across various regions of the mouse brain. (**D**) Validation of the Rem2 antibody by Western blotting and immunohistochemistry. Left, Representative image of Western blot using striatal lysates from wildtype and Rem2 knockout mice with β-actin serving as a loading control. Right, Representative coronal sections from wildtype and Rem2 knockout mice showing Rem2 immunoreactivity in magenta and DAPI counterstain in blue. Scale bars are 500 μm in all images. GP, globus pallidus; IPAC, interstitial nucleus of the posterior limb of the anterior commisure; NAcc; nucleus accumbens; OT, olfactory tubercle; STR, striatum; SNr, substantia nigra reticulata; VP, ventral pallidum.

Next, we characterized Rem2 protein expression by capillary electrophoresis followed by immunodetection (Fig. 2A,B) and by immunofluorescence (Fig. 2C). For protein quantification, 16 nervous system tissues were collected and analyzed (Fig. 2A). Similar to mRNA expression, Rem2 protein was relatively abundant in the striatum, trigeminal ganglion and pituitary. Interestingly, Rem2 protein was also abundant in the globus pallidus and substantia nigra, areas where Rem2 mRNA expression was negligible. Rem2 immunofluorescence was consistent with the capillary electrophoresis data, confirming that Rem2 is expressed within multiple nuclei of the basal ganglia, extended amygdala, and in the CA1 and CA3 regions of the hippocampus. To confirm the specificity of the Rem2 antibody, a Western blot was performed using striatal lysates from Rem2 knockout and wildtype mice. As shown in Fig. 2D, the band corresponding to Rem2 protein was absent in the knockout sample. We also preformed IHC on sections from Rem2 knockout and wildtype mice and Fig. 2D shows a lack of Rem2 immunoreactivity at the level of the striatum. Additionally, Rem2 immunoreactivity presented in Fig. 2C was absent in the knockout animal (data not shown).

### Rem2 is expressed in striatal MSNs, but not in interneurons

**Figure 3:**
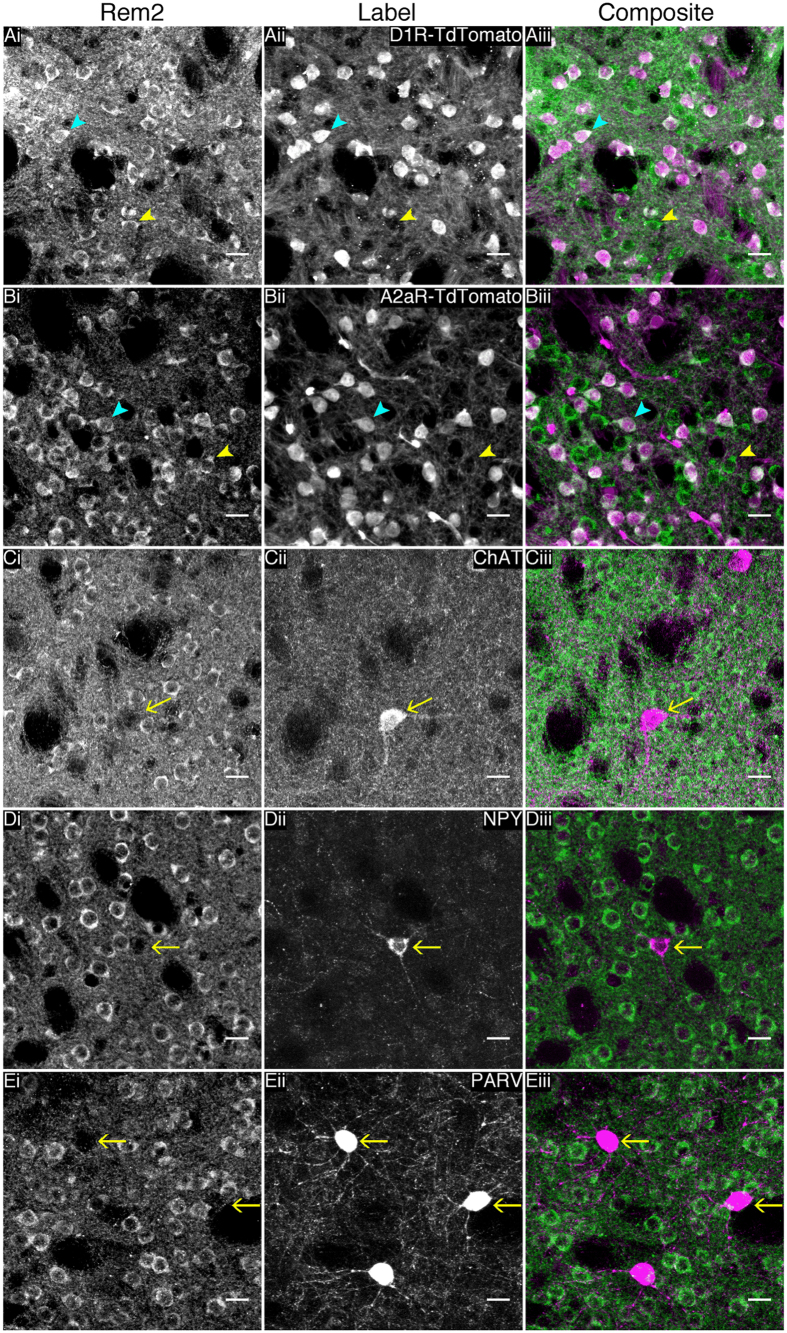
Rem2 is expressed in striatonigral and striatopallidal medium spiny neurons, but not striatal interneurons. (**A,B**) Representative images of Rem2 immunofluorescence on striatal sections from Cre dependent td-tomato reporter mice that express Cre recombinase under the dopamine D1 receptor or adenosine A2a receptor promoter, respectively. Blue arrowheads indicate cells expressing Rem2 and td-tomato, while yellow arrowheads indicate cells expressing Rem2 but not td-tomato. (**C–E**) Representative images of double-label immunofluorescence for Rem2 and choline acetyltransferase (**C**), neuropeptide Y (**D**) or parvalbumin (**E**), show that Rem2 is not expressed in any striatal interneurons examined. Yellow arrows indicate the cell bodies of interneurons that do not colabel with Rem2. ChAT, choline acetyltransferase; PARV, parvalbumin; NPY, neuropeptide Y. Scale bars are 20 μm and the same in all images.

Since Rem2 mRNA and protein was abundantly expressed in the basal ganglia, we performed a series of immunofluorescence and co-labeling experiments to determine which neuronal populations express Rem2. These studies were limited to the striatum because there was no evidence of somatic immunoreactivity in other nuclei of the basal ganglia. To examine Rem2 protein expression in MSNs of the striatonigral (D1R expressing MSNs) and striatopallidal (A2aR expressing MSNs) pathways, we performed immunofluorescence on the progeny of matings between Cre recombinase dependent td-Tomato reporter mice and D1R-Cre or A2aR-Cre mice (see materials and methods for details). Rem2 immunofluorescence co-labeled with cells expressing td-tomato in striatal sections from both D1R and A2aR reporter lines (Fig. 3A,B). Rem2 was also expressed in td-tomato negative cells in both D1R and A2aR reporter lines, which appeared to be striatopallidal and striatonigral MSNs, respectively. Additional co-immunofluorescence experiments in wildtype animals showed that Rem2 is not expressed in three major populations of striatal interneurons examined including large aspiny cholinergic interneurons, NPY expressing GABAergic interneurons and parvalbumin expressing GABAergic interneurons (Fig. 3C–E). Noteworthy, Rem2 protein appeared to be nuclear excluded, in contrast to Rem2 localization in primary cell cultures^6^,^8^,^19^.

### A subpopulation of large diameter sensory neurons expresses Rem2

**Figure 4:**
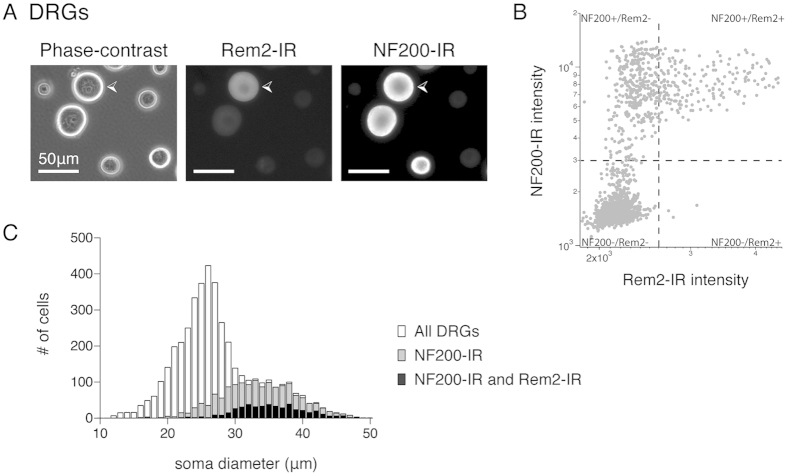
Rem2-immunoreactivity in dissociated dorsal root ganglion (DRG) neurons. (**A**) Representative DRG neurons under phase-contrast (left) and following immunocytochemistry (ICC) staining and fluorescence detection using Rem2 (middle) or Neurofilament 200 (NF200, right) antibodies. White arrowhead indicates a DRG neuron that displays positive-immunoreactivity against Rem2 and NF200 antibodies. Scale bar is 50 μm and is the same for all images. (**B**) Scatterplot of fluorescence intensity of DRG neurons stained for both NF200 and Rem2 from 1 experiment. Regions of interest were automatically generated around dissociated DRG neurons from the phase-contrast image and fluorescence intensity was measured from subsequent images collected from the Alexa Fluor® 488 or 568 channel. Each dot represents an individual DRG neuron. Dashed lines indicate the boundary between negative and positive-immunoreactivity as determined by staining in negative controls (secondary antibodies alone). (**C**) Distribution of DRG soma diameter from ICC experiments. White bars indicate the distribution of all DRG neurons (mean = 28.3 ± 0.1 μm, n = 4806, from 4 biological replicates) detected from phase-contrast images. Grey bars indicate the population of DRG neurons positive for NF200-immunoreactivity (mean = 34.5 ± 0.1 μm, n = 1675, from 4 biological replicates). Black bars indicate the population of DRG neurons positive for both NF200 and Rem2-immunoreactivity (mean = 36.5 ± 0.2 μm, n = 501, from 4 biological replicates).

Since *Rem2* mRNA was detected in DRG and TG samples by qPCR, the presence of Rem2 protein in DRG and TG neurons was assessed by immunocytochemistry. Immunoreactivity against Rem2 was detected in dissociated DRG neurons (Fig. 4A). Cell counts showed that 11% of all DRG neurons were Rem2 positive (782/7271 DRG neurons from 5 biological replicates) and Rem2-labeled neurons were a subpopulation of large diameter DRG neurons (mean diameter = 36.2 ± 0.2 μm, n = 782 from 5 biological replicates). Co-labeling with a marker of myelinated A-fibers, Neurofilament 200 kD (NF200), showed that 97% of Rem2 positive DRG neurons co-expressed NF200 (501/514 from 4 biological replicates) and 30% of all NF200 positive DRG neurons also expressed Rem2 (501/1675 from 4 biological replicates). Figure 4C illustrates the distribution of DRG neurons by soma diameter and confirms the population of DRG neurons positive for both NF200 and Rem2 are a subset of large diameter DRG neurons.

Similar results were obtained while examining dissociated TG neurons for Rem2-immunoreactivity. A subpopulation of large diameter TG neurons expressed Rem2 (mean diameter = 32.8 ± 0 μm, n = 1462 TG neurons from 4 biological replicates) and virtually all Rem2 positive neurons co-labeled with NF200 (99%, 1442/1462 Rem2+ve TG neurons from 4 biological replicates). Interestingly, compared to DRG neurons, a larger proportion of TG neurons expressed Rem2 (29%, 1462/5247 total TG neurons from 4 biological replicates) and a larger proportion of NF200 positive neurons co-labeled with Rem2 (56%, 1442/2573 NF200+ve TG neurons from 4 biological replicates).

### Post-translational modification of Rem2 by phosphorylation

**Figure 5:**
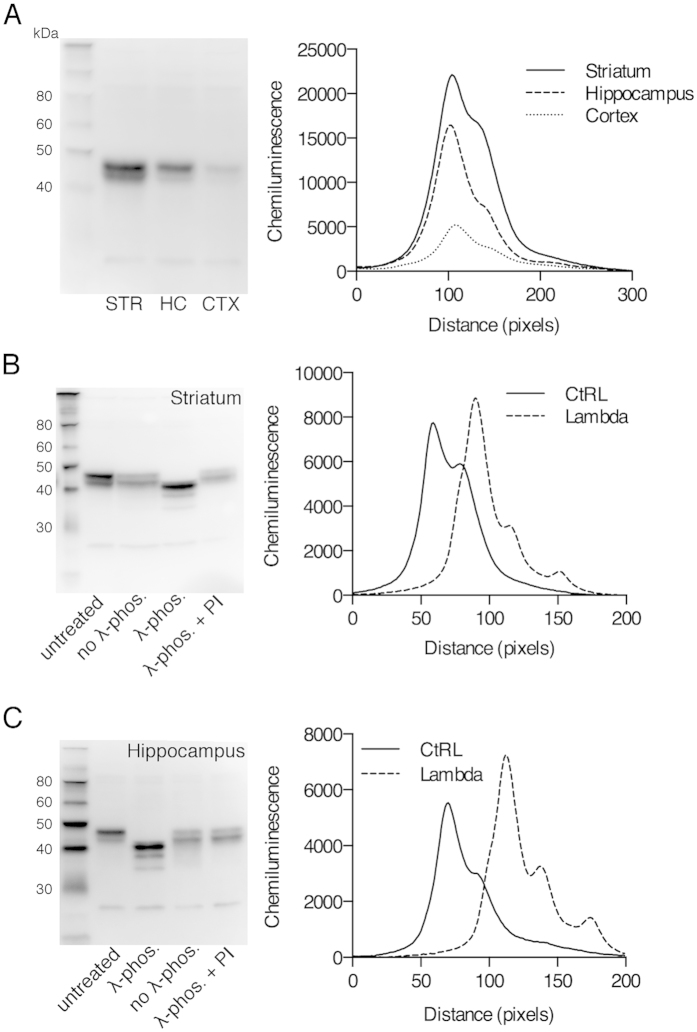
Post-translational modification of Rem2 by phosphorylation. (**A**) Representative Rem2 western blot (left) using sample lysates from striatum (STR), hippocampus (HC), and cortex (CTX), and chemiluminescence measurements of each lane (right). Line measurements represent the average signal across the entire width of each lane. (**B**) Representative Rem2 western blot of striatum lysates used in dephosphorylation experiment (left) and corresponding line graphs of chemiluminescence (right). Striatum lysates were untreated, treated with lambda phosphatase (λ-phos.), treated without lambda phosphatase (no λ-phos.), or treated with lambda phosphatase and phosphatase inhibitors (λ-phos.+PI). (**C**) Representative Rem2 western blot of hippocampus lysates used in dephosphorylation experiment (left) and corresponding line graphs of chemiluminescence (right). Lane labels are as described in (**B**). Images are representative of at least 3 independent experiments.

Native Rem2 protein runs as a doublet under traditional denaturing western blot conditions (Fig. 5A), an observation also observed using embryonic cortical lysates^8^, suggesting that Rem2 is subject to post-translational modification. In heterologous expression systems, RGK proteins are subject to phosphorylation, which modifies their protein-protein interactions and ultimately their physiological function^13^,^20^. Therefore, using a validated antibody for native Rem2 protein, we investigated the phosphorylation state of Rem2 using protein extracts from striatum and hippocampus. Samples were treated with lambda phosphatase and analyzed by Western blotting. Treatment with lambda phosphatase collapsed the Rem2 doublet (λ-phos. lane) in samples from striatum (Fig. 5B, representative of 3 or more replications) and hippocampus (Fig. 5C, representative of 3 or more replications). As a control, two additional reactions were run; one without lambda phosphatase (no λ-phos.) and one with lambda phosphatase plus phosphatase inhibitors (λ-phos + PI). Quantification of the chemiluminescence signal from lanes labeled untreated and λ-phos., using line measurements, confirmed that treatment with lambda phosphatase collapsed the Rem2 doublet. The line measurements show that in addition to collapsing of the Rem2 doublet, the subsequent collapsed band, representing full length Rem2 protein, migrated further then either of the original Rem2 bands. Noteworthy, dephosphorylation appeared to destabilize the Rem2 protein, resulting in the appearance of two lower molecular weight bands.

### Developmental expression of Rem2

**Figure 6:**
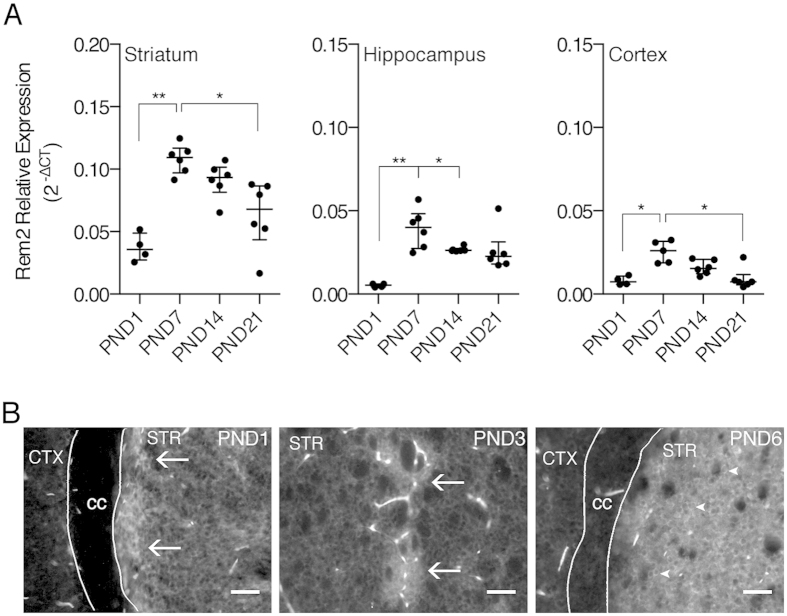
Developmental expression of Rem2 mRNA and protein. (**A**) Postnatal expression of *Rem2* mRNA in striatum, hippocampus and cortex. Total RNA was isolated from the indicated brain regions at postnatal day (PND) 1 (n = 4), 7 (n = 6), 14 (n = 6), and 21(n = 6), and *Rem2* mRNA was quantified by qPCR. *Rem2* gene expression was normalized to the geometric mean of the three “housekeeping” genes, *Actb*, *Gapdh*, and *Tbp. Rem2* gene expression varied significantly across postnatal development in all brain regions examined. *p < 0.05 and **p < 0.01, Kruskal-Wallis test followed by Dunn’s multiple comparisons test. (**B**) Representative images of Rem2 immunofluorescence in dorsal striatum at PND1, PND3, and PND6. Arrows in PND1 and PND3 images indicated patchy enrichment of Rem2 protein and arrowheads in PND6 images indicate somal rim-like expression. Noteworthy, the bright structures present in the images represent auto-fluorescent blood vessels. Scale bars are 50 μm and the same in all images. cc, corpus callosum; CTX, cortex; STR, striatum.

Rem2 has been implicated in development of the nervous system in zebrafish^9^ and identified as a protein involved in synaptogenesis^7^. Therefore, we quantified the amount of *Rem2* mRNA at time points spanning postnatal synaptogenesis in the mouse (Fig. 6A). In the striatum, *Rem2* mRNA was differentially expressed over the time frame examined (K = 16.09, p < 0.01). *Rem2* mRNA peaked at PND7 (PND7 vs PND1, p < 0.01) and gradually declined over the next two weeks (PND7 vs PND21, p < 0.05). Similar results were obtained in the hippocampus (K = 14.14, p < 0.01) and cortex (K = 12.15, p < 0.01). In the hippocampus, *Rem2* mRNA peaked at PND7 (PND1 vs PND7, p < 0.01), and remained elevated to at least PND14 (PND1 vs PND14, p < 0.05). In the cortex, *Rem2* mRNA also peaked at PND7 (PND7 vs PND1, p < 0.05) and, as in the striatum, declined over the next two weeks (PND7 vs PND21, p < 0.05). Notably, the overall relative levels of *Rem2* transcript across brain regions were similar to that observed in adult animals.

Neonatal Rem2 expression was also assessed in the striatum using immunofluorescence (Fig. 6B). At PND1 and PND3, Rem2 protein expression was detected in the striatum with enrichment in distinct patches in the dorsal striatum and along the medial boarder of the corpus callosum. By PND6, Rem2 protein expression appeared more homogeneous throughout the striatum. This temporal pattern of expression is consistent with postnatal maturation of the striatum, with striosome, or dopamine “island”, development preceding the matrix compartment^21^. Interestingly, at PND6, Rem2 protein appeared to partially relocalize as Rem2 immunoreactivity was apparent in cell bodies; in contrast to PND1 and PND3, where Rem2 immunoreactivity was present in the striatum, but the cellular localization was unclear. Additionally, similar to the findings in adult animals, the expression pattern in the soma appeared rim-like, which is suggestive of nuclear exclusion. Noteworthy, the structures with saturated signal represent auto-fluorescent blood vessels, which was unavoidable because early postnatal pups were too small for transcardial perfusion techniques.

## Discussion

Rem2 is a member of the RGK family of small GTPases. Heterologous expression of Rem2 inhibits HVA calcium channels^1^,^2^,^3^,^4^ although the physiological significance of this action is unknown. In addition, Rem2 has been implicated in regulating dendritic arborization and synapse formation in central neurons^6^,^7^. Rem2 is reported to be the primary RGK expressed in nervous system tissues, however, there is little information on the precise expression patterns of Rem2 within the nervous system. Therefore, in the current study, we have characterized *Rem2* mRNA and protein expression throughout the nervous system, identified specific central and peripheral neuronal populations expressing Rem2 protein, and characterized Rem2 expression across a timeframe of postnatal neuronal maturation. This study will be useful for uncovering physiological functions of Rem2.

We examined *Rem2* gene expression in the CNS using tissue arrays (Fig. 1A). Although qPCR amplification was observed in every sample examined (except liver), *Rem2* mRNA expression was enriched in specific regions. In the CNS, *Rem2* mRNA was most abundant in the striatum and CA3 region of the hippocampus, with relatively moderate levels in several other regions including the amygdala, hippocampus and BNST. Although the approach used in this study limited quantification of *Rem2* mRNA to large nuclei or collections of nuclei (i.e. thalamus and midbrain), our results are consistent with *in situ hybridization* data presented by a previous report that identified *Rem2* as a gene enriched in nuclei of the central extended amygdala^18^. We also examined *Gem* expression (Fig. 1B) because two studies reported functions of Gem in DRG neurons^16^,^17^. *Gem* mRNA was negligible in DRGs and in the majority of other tissues examined except for the spinal cord and pituitary gland. Although *Gem* expression was low in DRGs, we cannot exclude the possibility that *Gem* expression is upregulated in a stimulus dependent manner as transcriptional regulation is a common feature of the RGK protein family^1^. To that point, a recent study found that transcription of *Rrad* increased in the hippocampus following kainic acid-induced status epilepticus, a response dependent on serum response factor^22^.

Results from our protein assay using capillary electrophoresis (Fig. 2A) are in general agreement with the qPCR data such that relatively high expression of Rem2 protein was found in the striatum and hippocampus and moderate levels in the amygdala. However, we also found high Rem2 protein expression in the globus pallidus and substantia nigra, regions with negligible *Rem2* mRNA expression. These data suggest that Rem2 expression is localized to afferent fibers projecting into these nuclei. Given that Rem2 mRNA and protein is most abundantly expressed in the striatum it is likely that axonal localization of Rem2 in striatal MSNs accounts for Rem2 expression in the aforementioned nuclei. Consistent with this interpretation, Rem2 protein was localized to MSNs of the striatopallidal and striatonigral pathways (Fig. 3).

We performed co-labeling experiments to identify neuronal populations that express Rem2 in the striatum (Fig. 3). As mentioned above Rem2 immunoreactivity co-localized with tdTomato in D1R- and A2aR-tdTomato reporter mice, demonstrating that Rem2 is expressed in MSNs. In addition, we performed immunofluorescence experiments with markers of striatal interneurons including choline acetyltransferase, neuropeptide Y and parvalbumin. Rem2 did not co-label with these markers, suggesting that Rem2 expression is restricted to MSNs, although we cannot completely exclude expression in rare and novel striatal interneurons^23^. We did not explicitly identify the neuronal populations expressing Rem2 in the hippocampus, but given the pattern of immunoreactivity in this region, it is likely that pyramidal neurons in CA3 and CA1 express Rem2. Consistent with this interpretation, Rem2 mRNA is localized to the pyramidal cell layers in the hippocampus^18^.

*Rem2* transcript and protein were also found in primary sensory ganglia (Fig. 4), including dorsal root and trigeminal ganglia. Rem2-expressing sensory neurons were large soma diameter neurons that stained positive for NF200 and thus likely corresponded to myelinated Aα afferents which innervate muscle fibers and convey proprioceptive information, or Aβ afferents, which respond to innocuous touch. This population of neurons has been shown to undergo changes in animal models of osteoarthritic and neuropathic pain^24^,^25^. Further work characterizing the Rem2-positive population and its functional role in sensory neurons is currently underway.

Expression levels of Rem2 were examined over the first three weeks of postnatal development (Fig. 6), a critical time window for neuronal maturation in the rodent. *Rem2* mRNA expression peaked at PND7 in the striatum, hippocampus and cortex and appeared to decline over the next two weeks. In the striatum, elevated mRNA expression correlated well with our immunohistochemistry results. In the early postnatal striatum (PND1 and PND3), Rem2 protein expression was observed as distinct patches and along the ventral tier of the corpus callosum, while by PND6 Rem2 expression was relatively high and uniform throughout the striatum. The restricted pattern of Rem2 expression observed at PND1-3 reflects the dopamine “islands” present in the perinatal striatum^21^, areas that give rise to the striosomes in mammals^26^. The preferential expression of Rem2 in striosomes at PND1 and PND3 might occur because MSNs in this compartment are ontogenetically ahead of the cells in the surrounding matrix^27^,^28^,^29^,^30^,^31^.

Although the physiological significance for a transient elevation of Rem2 in the brain is unknown, it may be related to postnatal dendritic and/or synaptic maturation. Knockdown of Rem2 in primary hippocampal or cortical neurons results in a decrease in the density of mature dendritic spines and functional synapses, and increases the complexity of the dendritic arbor^6^,^7^. In the mouse striatum, the MSN dendritic arbor undergoes significant remodeling between PND7 and PND14^32^. For example, over this timeframe, the mean dendritic length increases, but the complexity of the arbor decreases. Additionally mature dendritic spines and synapses begin to appear around PND7 and increase over the next two weeks^32^,^33^,^34^. Thus, the observed increase in postnatal Rem2 expression coincides with neuronal maturation in the rodent brain.

We can only speculate on the mechanism responsible for a transient increase in *Rem2* mRNA, but it could be related to striatal activity during development. Experiments have shown that dendritic spine formation and synaptogenesis in the striatum is driven by network activity in the basal ganglia^35^. Authors of this study demonstrated that activity of corticostriatal afferents between PND8-15 is required for spine formation and synaptogenesis. Thus it is conceivable that excitatory drive onto MSNs results in activity dependent transcription of *Rem2*. This hypothesis is supported by data demonstrating activity dependent transcription of *Rem2* in primary cell cultures and following light stimulation in *X. laevis* tadpoles^5^.

Although RGK proteins contain a core domain similar to other monomeric GTPases, they do not have many of the characteristic features of a GTPase^2^,^4^. For example, minimal changes are observed in the crystal structures of the Rem2 core domain between GDP and GTP bound states^10^. Also, Rem2 displays relatively low intrinsic GTPase activity and no associated GEFs or GAPs have been identified to date^11^. It is proposed that RGK proteins may not operate using the canonical mechanism of guanine nucleotide cycling. Thus, it is likely that Rem2 is regulated by other mechanisms such as phosphorylation, as shown in previous studies^8^,^13^. We performed traditional gel electrophoresis and found that Rem2, isolated from adult brain tissues, migrates as a doublet (Fig. 5). Further, the doublet collapsed following phosphatase treatment, demonstrating that Rem2 is subject to post-translational phosphorylation. Our line measurements show that the collapsed Rem2 band migrated further than either of the two parent bands, suggesting that Rem2 is phosphorylated at multiple residues. Although, we are proceeding with this interpretation cautiously as two putative break down products were observed following phosphatase treatment.

The identity of the phosphorylated residues is unknown, however there are likely candidates. An unbiased proteomic study identified S334 as a phosphorylated site in mouse brain^36^. Interestingly, phosphorylation of this site in addition to S69 is required for Rem2 association with 14-3-3 proteins^13^. If Rem2 is in fact phosphorylated at multiple residues as our data suggests, it is possible that Rem2 exists bound to 14-3-3 proteins under basal conditions. Hypothetically, 14-3-3 may sequester Rem2 and dephosphorylation of either S334 or S69 could release Rem2 to allow interactions with other binding partners, such as the beta subunits of HVA calcium channels. Similar inhibitory functions of 14-3-3 proteins have been identified in relation to other proteins^37^. Phosphorylation may occur at other residues, possibly by PKA or CaMKIIα^8^,^13^, and it is likely that Rem2 phosphorylation is dependent on cellular context.

In conclusion, we have shown that Rem2 is expressed in a variety of nervous system tissues, but is enriched in the basal ganglia. The function of Rem2 in the basal ganglia is unknown, but may be important for neuronal maturation during development, and/or neuroplasticity in adulthood through interactions with HVA calcium channels.

## Materials and Methods

### Animals

All animal studies were conducted in accordance to the National Institutes of Health’s *Guidelines for Animal Care and Use* and all experimental protocols were approved by the National Institute of Alcohol Abuse and Alcoholism Animal Use and Care Committee. Male and female wildtype or mutant C57BL/6 mice (>30 days, unless otherwise noted) were used for experiments. To examine Rem2 colocalization with striatonigral MSNs, reporter mice were generated by mating heterozygous B6.Cg-Tg(Drd1a-cre)^217Gsat/Mmcd^ mice with homozygous B6.Cg-Gt(ROSA)^26Sortm14(CAG-tdTomato)Hze^/J mice. To examine Rem2 colocalization with striatopallidal MSNs, reporter mice were generated by mating heterozygous B6.Cg-Tg(Adora2a-Cre)^KG139Gsat/Mmucd^ mice with homozygous B6.Cg-Gt(ROSA)^26Sortm14(CAG-tdTomato)Hze^/J mice.

A conditional Rem2 knockout mouse line (B6.Cg-Rem2^tm3551(T2A-mkate2)Arte^) was generated by homologous recombination (TaconicArtemis) using a targeting vector constructed with BAC clones from the C57BL/6J RPCIB-731 BAC library. The targeted Rem2 allele contained sequences for the open reading frame of mKate2 and T2A, which were inserted between the 5′ UTR and translation initiation codon. LoxP sites were inserted to flank a 1.6 kb region containing exons 2 and 3 of the Rem2 gene. The targeting vector was transfected into TaconicArtemis C57BL/6N Tac stem cells and homologous recombinant clones were selected for blastocyst injection. Mutant mice generated were backcrossed onto the C57BL/6J strain for 2 generations prior to antibody validation experiments. The Rem2 antibody used in these experiments was validated using global Rem2 knockout mice. Global knockout mice were generated by inbreeding the F1 generation from a Rem2^LoxP/LoxP^ X B6.C-Tg(CMV-cre)^1Cgn^/J (Jackson Laboratories, SN# 006054) cross.

### qPCR

Mice (P56–P60, unless otherwise noted) were anesthetized with isofluorane and euthanized by rapid decapitation prior to tissue harvesting. Coronal sections (250 μm thick) were prepared in ice-cold modified aCSF (sucrose,194 mM; NaCl, 30 mM; KCl, 4.5 mM, MgCl, 1.0 mM; NaHCO_3_, 26.0 mM; NaH_2_PO_4_, 1.2 mM; Glucose, 10 mM) bubbled with a 5% CO_2_/95% O_2_ gas mixture and regions of interest were microdissected bilaterally and placed in Trizol^®^ (Invitrogen) for isolation of total RNA. RNA was isolated using the RNeasy^®^ Lipid Tissue Mini Kit (Qiagen) according to the manufacturers protocol and RNA quality and quantity was assessed using RNA 6000 Nano Kits on a Bioanalyzer 2100 (Agilent). RNA quality was determined using the RNA integrity number^38^; these values were typically above 8.0. RNA (100–200 ng) was reverse transcribed using the QuantiTect^®^ Reverse Transcription Kit (Qiagen) according to the manufacturer’s protocol. qPCR was performed using a StepOnePlus^TM^ system, predesigned TaqMan^®^ Gene Expression Assays, and TaqMan^®^ Universal Master Mix II (all from ThermoFisher Scientific). Singleplex reactions contained 1 μL of the predesigned gene expression assay (*Rem2*, Mm00600529_m1*; Actb, 4352933E; Gapdh*, 4352339E; *Tbp*, Mm01277045_m1), 2 μL of cDNA, 10 μL of master mix and 7 μL of RNase/DNase-free water. qPCR was performed according to the manufacturer’s recommended settings: 50 °C for 5 min, 95 °C for 10 min and then 40 cycles of melting at 95 °C for 15 sec and annealing/extension at 60 °C for 1 min. Reactions were run in triplicate for at least 4 biological replicates per group (except spinal cord, which contained 3 biological replicates). PCR plots were analyzed by the 2^−ΔCT^ method^39^. The C_T_ value was defined as the cycle number at which the amplification curve reached a ΔRn (Rn − baseline, where Rn is the fluorescence of the reporter dye divided by the fluorescence of a passive reference dye) threshold set at 0.1. For quantification of relative *Rem2* and *Gem* gene expression in the tissue array study presented in Fig. 1, *Actb* was used as an internal control so that ΔC_T_ = C_T_ (*Rem2 or Gem)* − C_T_ (*Actb*). For quantification of relative *Rem2* gene expression in the development study presented in Fig. 6, the geometric mean of multiple internal controls was calculated^40^ so that ΔC_T_ = C_T_(*Rem2*) − 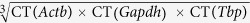.

### Protein expression

Tissue samples were collected from adult mice (P56 – P60) as described for qPCR experiments, however, following microdissection, samples were flash frozen on dry ice and stored at −80 °C until processing. Tissue samples were homogenized using RIPA buffer (ThermoFisher Scientific) containing Halt^TM^ protease and phosphatase inhibitor cocktail (ThermoFisher Scientific), centrifuged at 13,000× g for 10 min at 4 °C, and the supernatant was collected for analysis. Total protein concentrations were determined using the Pierce^®^ BCA Protein Assay Kit. Rem2 protein expression was quantified using a Wes^TM^ capillary electrophoresis system (ProteinSimple^®^) set to the following parameters: stack loading time 15 sec; sample load time 9 sec, separation time 25 min, primary antibody incubation time 30 min and secondary antibody incubation time 30 min. Based on preliminary experiments, assays were run using 0.5 μg/μL denatured total protein, goat anti-Rem2 (Santa Cruz Cat# sc-160722; 200 μg/mL diluted at 1:50) primary antibodies, and HRP-conjugated mouse anti-goat (Thermo Scientific Cat# 31430; 400 μg/mL diluted at 1:100) secondary antibodies. Chemiluminescence was imaged at exposure times ranging from 30 sec to 960 sec and a virtual time = 0 sec was extrapolated using Compass software (ProteinSimple®) to create a “multi-image” electrophoretogram. Rem2 protein expression was quantified by integrating the peak corresponding to the Rem2 signal. Initially, Rem2 signal was normalized to β-actin, however significant variation in β-actin abundance across brain regions was observed. Therefore, the Rem2 signal was normalized to total protein.

### Immunohistochemistry (IHC)

Mice were anesthetized with isofluorane and transcardially perfused with 20 mL of 0.1 M phosphate buffered saline (PBS, pH 7.4) followed by 20 mL of 4% formaldehyde in PBS. Following perfusion, brains were extracted and post-fixed in 4% formaldehyde overnight at 4 °C (except for a brain that was used for choline acetyltransferase (ChAT)/Rem2 co-immunofluorescence, which was post-fixed for 2 hr). Brains were serial sectioned (1:6 for adult brains and 1:3 for neonatal brains) at 40 μm on the coronal plane from approximately +4.0 mm to −4.5 mm relative to the bregma and stored in cryoprotectant (30% glycerol and 30% ethylene glycol in 0.1 M phosphate buffer, pH = 7.4) at −20 °C until further processing. Sections were removed from cryoprotectant and rinsed 3 × 5 min in PBS. Next, sections were rinsed in ddH_2_0 for 5 min and incubated in sodium borohydride (5 mg/mL) to reduce autofluorescence^41^ and then rinsed 3 × 5 min in PBS. Sections (except those for ChAT/Rem2 co-immunofluorescence) were then antigen retrieved in sodium citrate buffer (10 mM sodium citrate, 100 mM sodium chloride, pH 6.0) for 1 h at 65 °C, followed by 3 × 5 min rinses in PBS. Next, sections were permeabilized and blocked for 1 h at RT using a blocking solution consisting of 5% bovine serum albumin (BSA) and 0.2% Triton-X 100 in PBS. Following blocking, sections were incubated in primary antibodies diluted in blocking solution for approximately 48 h at 4 °C. The following primary antibodies were used for IHC experiments: Goat anti-Rem2 (Santa Cruz Cat# sc-160722; 200 μg/mL affinity purified polyclonal diluted at 1:500), Rabbit anti-ChAT (Millipore Cat# AB143; polyclonal antisera diluted at 1:1000), Rabbit anti-neuropeptide Y (Immunostar Cat# 22940; polyclonal antisera diluted at 1:1000), Rabbit anti-parvalbumin (Swant^®^ Cat# PV 27; polyclonal antisera diluted at 1:500). After primary antibody incubation, sections were rinsed 3 × 20 min in blocking solution and transferred to Alexa Fluor^®^-568 donkey anti-Rabbit (1:1000) and/or Alexa Fluor^®^-488 donkey anti-goat (1:1000) secondary antibodies for 1 h at RT. Finally, sections were rinsed 2 × 20 min in PBS w/0.2% Triton-X 100 (PBS-T) and 1×20 min in PBS. Following IHC, sections were mounted and coverslipped with No. 1.5 cover glass and Fluoromount G (RI = 1.40, Electron Microscopy Sciences). Low magnification (5×) epifluorescence images were acquired with a Zeiss Axiovert 200 microscope equipped with a Zeiss Axiocam MR monochrome CCD camera and AxioVision acquisition software (v4.8). For co-immunofluorescence imaging, a Zeiss LSM 510 META confocal microscope equipped with DPSS and Argon/Krypton lasers for 561 nm and 488 nm excitation, respectively, and a Zeiss C-Apochromat 40x water immersion objective (1.20 NA) was used. Images were acquired with the pinhole adjusted to 1.0–2.6 AU. Optical sections (1.0–2.5 μm) were acquired and the resulting z-stacks converted to maximum intensity projections for display. ImageJ software (v.1.48) was used to adjust contrast of images for presentation in figures.

### Dorsal root ganglia (DRG) and trigeminal ganglia (TG) dissection and dissociation

Male or female adult mice were anesthetized by CO_2_ inhalation and decapitated before dissection. The spinal column was isolated and a laminectomy was performed to expose the dorsal side of the spinal cord. The spinal cord was removed by cutting attached dorsal roots. DRG were located by following dorsal root branches to intervertebral foramina, carefully dissected and placed in chilled Hanks balanced salt solution. TG were located at the base of the skull following removal of the brain and were carefully removed and placed in chilled Hanks balanced salt solution. To dissociate into individual neurons, DRG or TG were transferred to a enzymatic solution consisting of 1.3 mg/mL collagenase (CLS4; Worthington Biochemical), 0.2 mg/mL trypsin (Worthington Biochemical) and 0.1 mg/mL DNase I in Earles’ balanced salt solution supplemented with 3.6 g/L D-glucose and 10 mM HEPES. Ganglia were incubated at 36 °C for 1 h in a water bath shaker rotating at 110 rpm. After incubation, neurons were mechanically dissociated by vigorously shaking the flask for 10 s. Neurons were centrifuged (60× *g* for 6 min) and resuspended in minimum essential medium supplemented with 10% fetal bovine serum and 1% antibiotics (MEM+/+, all from Invitrogen) twice before being plated on polyethylenimine (PEI)-coated tissue culture dishes. Cells were maintained in a humidified 95% air/5% CO_2_ incubator at 37 °C before experiments.

### Immunocytochemistry (ICC)

Dissociated sensory neurons, 2–3 h after plating on tissue culture dishes, were washed with PBS before fixing with 2% formaldehyde for 30 min at RT. After washing out fixative with PBS, neurons were permeabilized with PBS-T for 20 min at RT. Following solution exchange with PBS, neurons were blocked with 4% BSA and 2% normal donkey serum in Tris-buffered saline (TBS) w/0.05% Tween-20 (TBS-T) for 1 h at RT. Primary antibodies, goat anti-Rem2 (1:500) and rabbit anti-Neurofilament 200 (NF200; Abcam, Cat# ab8135, 1:2000) in blocking solution were incubated with cells overnight at 4 °C. After washing cells with TBS-T for 1 h, cells were incubated with Alexa Fluor®-conjugated secondary antibodies (donkey anti-goat or donkey anti-rabbit, 488 nm or 568 nm, Molecular Probes, 1:2000) for 2 h at RT. Cells were washed with TBS-T for 1 h and then exchanged for PBS before imaging. All staining experiments included parallel negative controls, which received the same treatment except without primary antibody incubation. To image ICC experiments, 50–100 phase-contrast and fluorescence images were acquired using a Zeiss AxioObserver Z1 microscope equipped with fluorescence and an Andor Clara CCD camera controlled by open-source Micro-Manager software^42^,^43^ (v.1.4.18). Filters for Alexa Fluor® 488 (472/30 nm excitation, 520/35 nm emission) and Alexa Fluor® 568 (562/40 nm excitation, 641/75 nm emission) were used and exposure times for each channel were kept consistent across all samples acquired for a given biological replicate. ImageJ software (v.1.48) was used to adjust contrast of images for presentation in figures. For analyzing ICC experiments, a custom-written program written with Igor Pro software (version 6, WaveMetrics) was used to automatically select regions of interest around neurons in phase-contrast images and fluorescence intensity was measured from subsequent fluorescence images.

### Western blotting and phosphatase treatment

Striatum, hippocampus and cortex samples were dissected in ice-cold Hank’s balanced salt solution and immediately homogenized with RIPA buffer containing either Halt^TM^ protease and phosphatase inhibitor cocktail or only protease inhibitors. To determine if Rem2 is phosphorylated under basal conditions, either 20 or 40 μg of protein was electrophoresed on a pH discontinuous gel followed by standard Western blotting procedures. Membranes were blocked for 30 min with 5% milk and incubated with Rem2 primary antibodies (1:1000) overnight at 4 °C. Membranes were washed 3 × 10 min with 5% milk in TBS-T, incubated with HRP-conjugated mouse anti-goat antibodies (1:10,000) in blocking solution for 1 h at RT, and then rinsed 2 × 10 min in TBS-T and 1 × 10 min in TBS. For visualization, membranes were wetted with HRP substrate (Thermo Scientific, Cat# 34095) and imaged using a Kodak 4000R image station. Dephosphorylation experiments were performed using Lambda (λ) Protein Phosphatase (New England BioLabs Inc., Cat# P0753) and striatal and hippocampal lysates. Reactions (30 μL) containing 75–100 μg of protein and 800 units of λ-phosphatase were incubated at 30 °C for 50 min. Control reactions were performed simultaneously, which either contained phosphatase inhibitors or no λ-phosphatase. Dephosphorylation was confirmed by western blotting as described above.

### Statistics

Data was analyzed using GraphPad Prism 6 (GraphPad Software, Inc.). All data are presented as mean ± SEM except for Fig. 6, which is presented as median and IQR. Data was analyzed using the Kruskal-Wallis test followed by the Dunn’s multiple comparisons test. For all tests, α was set at 0.05.

## Additional Information

**How to cite this article**: Liput, D. J. *et al*. Rem2, a member of the RGK family of small GTPases, is enriched in nuclei of the basal ganglia. *Sci. Rep*. **6**, 25137; doi: 10.1038/srep25137 (2016).
